# Evaluation of Haplotype Inference Using Definitive Haplotype Data Obtained from Complete Hydatidiform Moles, and Its Significance for the Analyses of Positively Selected Regions

**DOI:** 10.1371/journal.pgen.1000468

**Published:** 2009-05-08

**Authors:** Koichiro Higasa, Yoji Kukita, Kiyoko Kato, Norio Wake, Tomoko Tahira, Kenshi Hayashi

**Affiliations:** 1 Division of Genome Analysis, Research Center for Genetic Information, Medical Institute of Bioregulation, Kyushu University, Fukuoka, Japan; 2 Division of Molecular and Cell Therapeutics, Medical Institute of Bioregulation, Kyushu University, Fukuoka, Japan; University of Oxford, United Kingdom

## Abstract

The haplotype map constructed by the HapMap Project is a valuable resource in the genetic studies of disease genes, population structure, and evolution. In the Project, Caucasian and African haplotypes are fairly accurately inferred, based mainly on the rules of Mendelian inheritance using the genotypes of trios. However, the Asian haplotypes are inferred from the genotypes of unrelated individuals based on population genetics, and are less accurate. Thus, the effects of this inaccuracy on downstream analyses needs to be assessed. We determined true Japanese haplotypes by genotyping 100 complete hydatidiform moles (CHM), each carrying a genome derived from a single sperm, using Affymetrix 500 K Arrays. We then assessed how inferred haplotypes can differ from true haplotypes, by phasing pseudo-individualized true haplotypes using the programs PHASE, fastPHASE, and Beagle. We found that, at various genomic regions, especially the MHC locus, the expansion of extended haplotype homozygosity (EHH), which is a measure of positive selection, is obscured when inferred Asian haplotype data is used to detect the expansion. We then mapped the genome using a new statistic, XDiHH, which directly detects the difference between the true and inferred haplotypes, in the determination of EHH expansion. We also show that the true haplotype data presented here is useful to assess and improve the accuracy of phasing of Asian genotypes.

## Author Summary

Precise haplotype maps are preferred for the performance of a variety of genetic studies including identification of disease-associated loci and dissection of evolutionary mechanisms such as selection and recombination. For diploid organisms, the haplotype information appears as the genotypes when we obtain the information using widely used high-throughput techniques. The process of extracting haplotype information from genotypes is called phasing, which can be accurately done if the genotypes are from related individuals, such as parent–child trios, by considering the constraints imposed by the rules of Mendelian inheritance. For the genotype data without family information, phasing is done by one of the methods that are based on haplotype clustering, and the inferred haplotypes are known to be less accurate. Here, we experimentally determined genome-wide definitive haplotypes using a collection of Japanese complete hydatidiform moles (CHM), each of which carries a genome derived from a single sperm. Using these resources, we asked if the definitive haplotype data can detect long-distance information that has been obscured when we rely solely on the haplotypes inferred by clustering. We also show that by introducing definitive haplotypes as references, inference of haplotypes of unrelated individuals is significantly improved.

## Introduction

Chromosomes carry records of mutational and recombinational events that they have experienced during their evolution as haplotypes. Thus, the exact knowledge of the genome-wide haplotype structure of a population can provide information essential to the study of the population history, and to association studies to discover alleles responsible for common diseases that are expected to have a long history since their first emergence. In this spirit, the International HapMap Project has been launched, and has achieved the construction of haplotype maps of extremely high resolution for three major populations: Africans, Asians, and Europeans [1],[2]. However, the accuracy of the haplotypes was not equal for the three populations, a fact that can affect the results of downstream studies.

Resolving haplotype structures from the available genotype data on the level of entire genomes remains a challenging task, both experimentally [3] and computationally [4]. Since the size of the datasets that researchers will want to phase is increasing dramatically, in terms of the numbers of both the loci and the individuals, the statistical estimation of haplotypes by phasing genotypes of randomly sampled individuals is potentially valuable, if it can be done accurately. Estimation by phasing has therefore received much attention in recent years, and several computational and statistical approaches have been developed [4]–[8]. Among these, the coalescence model and haplotype clustering have generally been accepted as reliable methods for inferring phases. However, even with these methods, haplotype estimation is not perfect in the absence of information from genetically related individuals [9]. In the International HapMap project, African (YRI) and European (CEU) haplotypes were inferred based mainly on family data (trios of children and their two parents). On the other hand, the East Asian (JPT+CHB) samples consisted of arbitrarily sampled individuals without any family data. Haplotypes that are inferred without trio information and based on relatively small samples may contain significant phasing errors (switch error) [10].

To avoid this issue of phasing uncertainty, we have experimentally determined genome-wide definitive haplotypes using complete hydatidiform moles [11],[12]. The formation of a CHM is started by a maternal event of rare sporadic occurrence: enucleation of the oocyte, followed by conjugation with a sperm, occurring in 0.1–0.05% of pregnancies. Thus, CHM genotypes are direct reads of sperm haplotypes that are unbiased and highly unlikely to be related to one another [13],[14], such that they can be considered to be randomly sampled from the population to which they belong [11], comparable to a population of lottery winners.

In previous work, we genotyped 74 Japanese CHMs with 280 K Perlegen SNPs [11],[12]. In the present study, we expanded our definitive haplotyping project of Japanese, by examining 100 CHMs using Affymetrix 500 K Array Sets. Using these data, we performed a genome-wide scan to detect significant differences between the definitive and inferred haplotypes. Our approach is a modification of the statistical method used to evaluate signals of positive selection from the extended haplotype homozygosity (EHH), first introduced by Sabeti et al. and further developed by others [15]–[18]. In this study, we compared integrated EHH of the same allele within a single population using different datasets, i.e., definitive and inferred haplotypes, and captured significant regional departures of this measure from background levels. We also show that the availability of definitive haplotypes can improve the accuracy of the inference of haplotypes of unrelated Asian genomes.

## Results

### Datasets and Their Nomenclature

The haplotype datasets of CHMs (this study) and the HapMap Project (CEU, CHB, JPT, and YRI) were used for comparison. The SNP allele calls of the Affymetrix Gene Chip Human Mapping 500 k Array Sets were used throughout, except where otherwise stated, because they were available in all the datasets. Two JPT samples, NA18987 and NA18992, whose estimated cryptic relatedness coefficients were more than 1/32 [2], were excluded from the analysis in order to avoid inappropriate inflation of the haplotype expansions due to their unusually long haplotype homozygosity [11].

To provide a comprehensive assessment of the haplotypes constructed by a variety of algorithms and samples, we prepared the following datasets, and named them using a “prefix-sample” rule. The prefix “PHASEd+” means that the parental haplotypes were inferred using the PHASE [4] program from the genotype data of trios, supplemented with pedigree information. “PHASEd-” means that the phasings were done by the same program from parental genotype data, but in the absence of pedigree information. “PHASEd+CEU”, “PHASEd+YRI”, and “PHASEd-JPT” were the corresponding haplotype datasets downloaded from the HapMap database (Release 22, corrected on June 30, 2008) [1] and extracted as the subsets for Affymetrix 500 K SNPs. In this study, some of the haplotypes of CEU parents were regionally inferred from their genotypes without trio information using PHASE (thus, “PHASEd-CEU”). The prefix “fastPHASEd-” means that the haplotypes were inferred from genotypes using the fastPHASE [8] program without pedigree information. The definitive haplotype dataset of CHMs lacks a prefix, since these haplotypes were determined directly, without inference. The prefixes were followed by the names of diploid genotype datasets, some of them carrying a second prefix, “pi.” This stands for “pseudo-individuals,” and indicates that the diploid datasets were made using randomly paired haplotypes that were directly determined (piCHM). The datasets and their names are summarized in Table 1.

**Table 1:** Summary of haplotype datasets.

Name	Details	Genotype Source	Phasing Done by
CHM	Directly determined haplotypes of 86 complete hydatidiform moles	This study	n.a.
fastPHASEd-piCHM	Haplotypes of CHMs inferred by fastPHASE after pseudo-individualizing CHM haplotypes	This study	This study
PHASEd-piCHM	Haplotypes of CHMs inferred by PHASE after pseudo-individualizing CHM haplotypes (chromosome 6p only)	This study	This study
PHASEd-JPT	Haplotypes of JPTs inferred by PHASE	HapMap	HapMap
PHASEd+CEU	Haplotypes of CEU parents inferred by PHASE with pedigree information	HapMap	HapMap
PHASEd-CEU	Haplotypes of CEU parents inferred by PHASE without pedigree information (chromosome 6p only)	HapMap	This study
fastPHASEd-CEU	Haplotypes of CEU parents inferred by fastPHASE without pedigree information	HapMap	This study
PHASEd+YRI	Haplotypes of YRI parents inferred by PHASE with pedigree information	HapMap	HapMap
fastPHASEd-YRI	Haplotypes of YRI parents inferred by fastPHASE without pedigree information	HapMap	This study

CHM, complete hydatidiform mole of Japanese collected by us; JPT, Japanese in Tokyo; CEU, Utah residents with Northern and Western European ancestry; YRI, Yoruba in Ibadan, Nigeria. JPT, CEU, and YRI are HapMap samples.

### Principal Component Analysis

We applied principal component analysis [19],[20] to our CHM datasets, together with those of the HapMap Project, in order to confirm the identity of each set and to detect possible hidden population structure in the samples. Figure S1 shows the plots for the first two eigenvectors calculated to resolve three Asian datasets (Figure S1A), or all five datasets of three ethnicities (Figure S1B), after filtering out SNPs with low call rates. The figure shows that both CHMs and JPTs were in the same cluster and were different from even the closest population, CHB, except for pseudo-individuals with the CHM065 haplotype (see also Figure S1C, Figure S1D, and Table S1). This haplotype seems to be of recent Asian ancestry, based on its coordinate. We excluded this haplotype from further analyses. After this exclusion, no substructure was discernible in the CHM dataset, and both CHMs and JPTs were seen to belong to the same population.

### Estimation of LD

Statistical estimates of LD from inferred haplotypes continue to be important for the purposes of defining a set of tagging SNPs [21],[22] and imputation-based approaches [23],[24] in association studies. To assess the accuracy of inferred haplotypes in estimating the LD measure, we compared the r^2^ calculated from the definitive haplotypes of CHM and phased piCHM using pairs of autosomal common SNPs (minor allele frequency >5%) within 200 kb regions. In this case, we employed fastPHASE, although it is known to be less accurate than PHASE, because of the limitations of our computational capacity.

**Figure 1:**
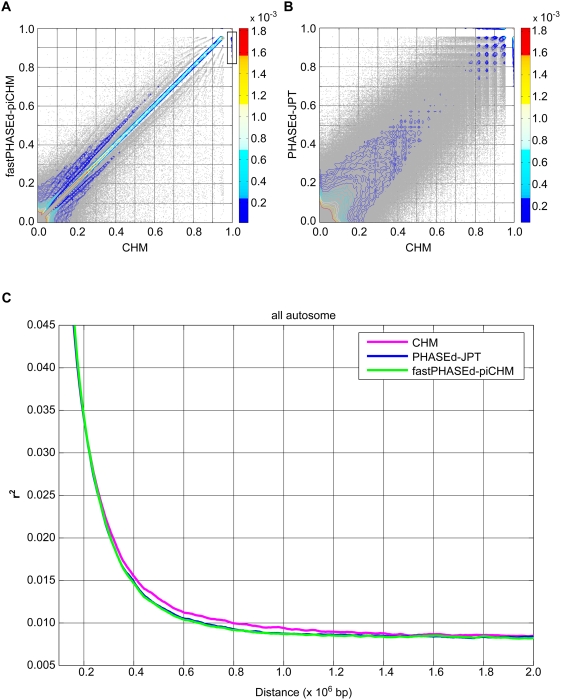
Comparison of r^2^ estimated from inferred and definitive haplotypes. r^2^ values for pairs of common SNPs (minor allele frequency >5%) within 200 kb sequences were calculated for haplotypes of CHM and fastPHASEd-piCHM (A), or CHM and PHASEd-JPT (B). The color ranges for fractions of pairs of 317,643 SNPs (A) and 327,408 SNPs (B) per area (0.01 r^2^ interval square) are indicated in a side bar. (C) The decay curves of r^2^ for all autosomal SNP pairs within 2 Mb are plotted against physical distance. The magenta, blue, and green lines denote the curves of the means of observed LD values in each distance (5 kb bin size) for CHM, PHASEd-JPT, and fastPHASEd-piCHM, respectively.

As shown in Figure 1A, even by phasing with fastPHASE, we found an extremely high concordance of r^2^ values between the two datasets (R = 0.991 for SNP pairs with r^2^>0.5), in accordance with earlier results [9],[25]. Marchini et al. have also assessed the accuracy of estimated r^2^ by root-mean-square-error (RMSE) measure after inferring haplotypes using simulated datasets. The RMSE between haplotypes of CHM and fastPHASEd-piCHM (0.0254) was higher than previous results of PHASE (0.011) for unrelated individuals, which probably is due to the difference of the phasing algorithms, or our real vs. their simulated datasets [9].

However, we also noted that a small but definite fraction of the estimations (boxed in the figure) revealed some deviation from strict correlation (tendency toward underestimation in phased dataset) even in the high r^2^ range (r^2^>0.8). Figure 1B shows the comparison of r^2^ values obtained from CHM and PHASEd-JPT. The values from the two datasets are again in good correlation (R = 0.952), other than the more scattered nature of the plot compared to Figure 1A (which is obviously because of sampling error due to the limited number of haplotypes contributing to each dataset). We again noticed that a small fraction of the estimates deviate from the strict correlation, similar to the observation in Figure 1A. No further analysis was done on these discrepancies of the r^2^ values, because they constitute an extremely small fraction and are unlikely to influence downstream analyses, such as imputations in association studies. However, the presence of these discrepant SNPs (or SNP pairs) may be indicative of some genomic regions that tend to be wrongly phased without Mendelian information.

A haplotype map is a crucial source of information not only for genome-wide association studies, but also for the detection of genomic regions possibly subject to selection. In the latter studies, the conservation of exceptionally long haplotypes is the indicator of positive selection, and long-distance LD structure can have significant effects. Therefore, we compared LD decay curves among these datasets (Figure 1C). As is evident from the figure, the mean r^2^ values of inferred haplotypes were consistently underestimated at long distances (>300 kb). Thus, we considered it important to reexamine possible positive selection using definitive haplotypes.

### Identification of Positively Selected Regions

Voight et al. have introduced a statistic (integrated haplotype score, iHS) to identify genomic regions of recent positive selection [17]. Their method is based on the detection of exceptional expansion of extended haplotype homozygosities (EHH), initially proposed by Sabeti et al. as an indicator of genomic region of positive selection [15].

**Figure 2:**
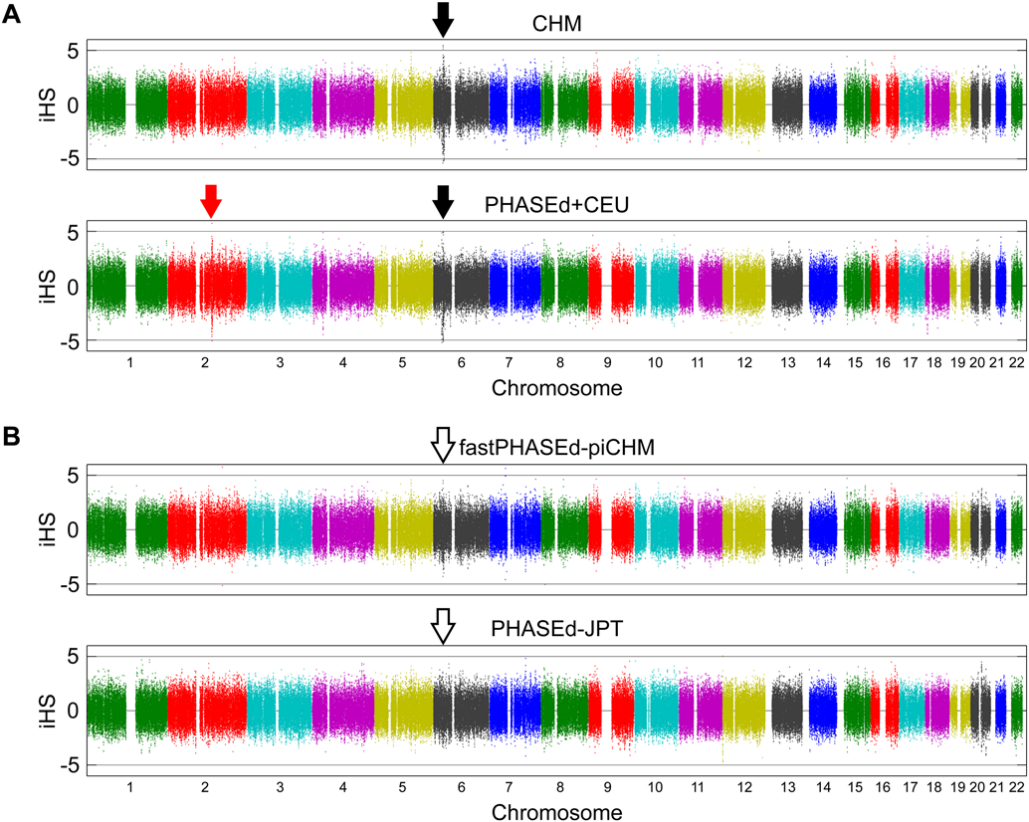
Genome-wide view of iHS. The iHS statistics for CHM (A), PHASEd+CEU (B), fastPHASEd-piCHM (C), and PHASEd-JPT (D) are plotted. Chromosomes are shown in alternating colors for clarity. The lactase gene and MHC loci are indicated with red and black arrows, respectively. See text for details.

We first carried out iHS analysis using CHM and PHASEd+CEU, both of which are virtually true haplotype datasets. As shown in Figure 2A, prominent iHS peaks were observed at several places, especially at the MHC locus (black arrows in the figure) in both CHM and CEU. Another peak, at the lactase locus (LCT), was found only in CEU. These, and other loci that show statistically significant iHS peaks, are listed in Tables S2 and S3.

Voight et al. have examined the haplotypes in HapMap populations by iHS analysis using SNP data of Phase I [17], and shown that several genomic regions are under positive selection, including the MHC locus at chromosome 6p. Their data indicate that this selection was significant for CEU and YRI, but not for JPT/CHB (See Table 2 in ref. [17]). Exceptional extension of long haplotypes in the MHC locus has also been reported by de Bakker et al. [26]. These authors also found that the extension was much more pronounced for CEU than for CHB or JPT, although this difference was not clearly stated in the text (see Figure 4 in ref. [26]). However, given the central role of this locus in the immune system, it is hard to interpret the population-dependent presence or absence of the selection. Rather, our results above suggest that the difference between populations is explained by the loss of information during the phasing of Asian samples without trio information.

We next examined fastPHASEd-piCHM and PHASEd-JPT (both are inferred Japanese haplotype datasets) by iHS, and the results are shown in Figure 2B. As is evident from the figure, the peak at the MHC locus was obscured in both cases, supporting the explanation that the information on expansion of EHH at this locus was lost during the inference of haplotypes.

Positive selection at the lactase locus has been documented in Caucasians and Africans but not in Asians [27],[28]. In this case, we also observed strong signal enrichment at this gene region only for CEU and not for CHM (Figure 2A, red arrow, and Table S2). Thus, the results confirm that the population-specific positive selection observed at the lactase locus is real.

Population-dependent positive selection was also examined by cross-population EHH (XP-EHH) [16] statistics using CHM vs. CEU and CHM vs.YRI datasets (Tables S4 and S5). As shown in the tables, many regions that had not been noted previously were newly identified. Some of these may have been missed in previous analyses [16] because of phasing problems. However, no definite conclusions could be reached, because the populations examined were not exactly the same.

### Mapping by Cross-Dataset Integrated Extended Haplotype Homozygosity

To more directly focus the lowering of information content in inferred haplotypes relative to definitive haplotypes, we introduce a new test; the cross-dataset integrated extended haplotype homozygosity (XDiHH). This statistic is similar to XP-EHH [16], except that the comparison is between two determinations of iHH: in the present case, inferred vs. definitive haplotypes. Here, iHH is an integration of EHH [17] against genetic distance, in both directions away from the core SNP, until at least two identical haplotypes are extended. XDiHH is expressed in simple terms by the following equation.
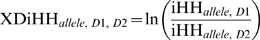
Here, *allele* is either ancestral or derived, depending on whether it is computed with respect to the ancestral or derived core allele, while *D1* and *D2* are the two datasets for comparison. When the rate of EHH decay is the same between datasets, XDiHH is equal to 0. Positive values indicate that *D1* haplotypes are longer than *D2* haplotypes, and negative values indicate the opposite.

In principle, phasing errors can result in false elongation or false shortening of extended haplotype homozygosity. Therefore, XDiHH can be both positive and negative. Simulation experiments using a simple two-SNP system and assuming various local r^2^ values indicate that XDiHH tends to decrease in high LD regions, while it tends to increase in low LD regions (see Figure S2 and its legend).

**Figure 3:**
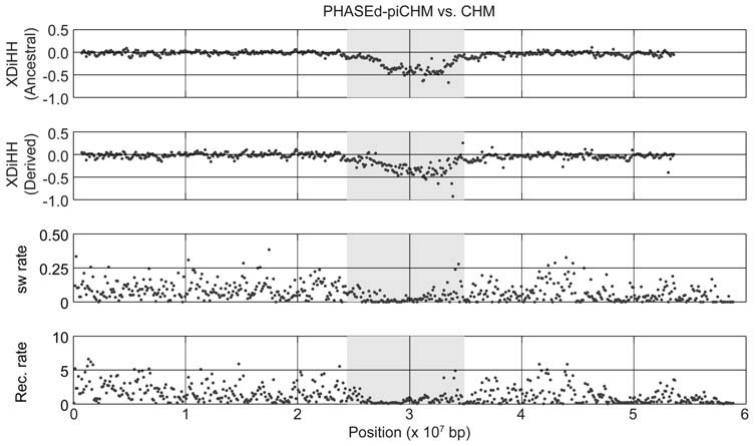
Fine-scale XDiHH map of chromosome 6p in CHM. XDiHH values of ancestral and derived alleles for PHASEd-piCHM vs. CHM are shown in the top two panels. The bottom two panels show the switch error (sw) rate, and recombination rate for the same 100 kb bin, respectively. The MHC locus is shown in gray. The recombination rate for the whole HapMap II population was obtained from HapMap [1],[31]. See text for details.

We examined the XDiHH statistics between the haplotypes of CHMs and those obtained by phasing of pseudo-individualized CHMs (piCHMs). Phasing of chromosome 6p was done by PHASE program (Figure 3). In this figure, we also plotted the switch error rate and recombination rate. The switch error rate was calculated as the proportion of neighboring heterozygous sites that are not correctly phased [29],[30]. The recombination rate was averaged over values for all HapMap populations, calculated from the data of HapMap II. As shown in Figure 3, we detected a negative signal at the MHC locus, corroborating the discrepancy of iHS results between fastPHASEd-piCHM and CHM.

It can also be seen in the figure that the switch error rate at MHC locus was low compared to the surrounding regions, which was contrary to naïve expectations, since the deviation of XDiHH from zero must be caused by switch errors. However, as described above, a consistent reduction of XDiHH is observed only if the switch error occurs in region of low recombination rate (i.e., high r^2^ regions), as demonstrated in Figure S2. Thus, the limited number of switch errors was sufficient to visibly reduce the XDiHH values on the MHC locus.

Essentially the same results were obtained using the CEU dataset of HapMap Phase II (Figure S3). That is, we used the parental genotype data of CEU [1] and constructed the haplotypes without offspring data using PHASE (that is, PHASEd-CEU) for chromosome 6p. We then compared these haplotypes with those from the HapMap database (that is, PHASEd+CEU) using XDiHH statistics. A prominent negative peak of XDiHH was also detected at the MHC locus (Figure S3), supporting the conclusion drawn based on observations of CHMs and piCHMs (Figure 3), that the negative XDiHH peaks at the MHC locus were the result of phasing in the absence of trio information.

Apart from MHC locus, the switch error rate of overall chromosome 6p region by PHASE was 0.0503 and 0.0633, for CEU without children and piCHM, respectively. These values are in accordance with the previously reported switch error rate of PHASE (0.0543), that was estimated by Marchini et al. [9] using randomly sampled genomic regions of HapMap CEU datasets removing the children.

### Genome-Wide Profile of XDiHH

We next asked if there are regions of expanded EHH, other than the MHC locus, that escaped detection when analyzed using inferred haplotypes. Because of the limitations of our computational capacity, we inferred the phases of piCHMs using fastPHASE. The loci that gave significant negative XDiHH values are listed in Table S6.

**Figure 4:**
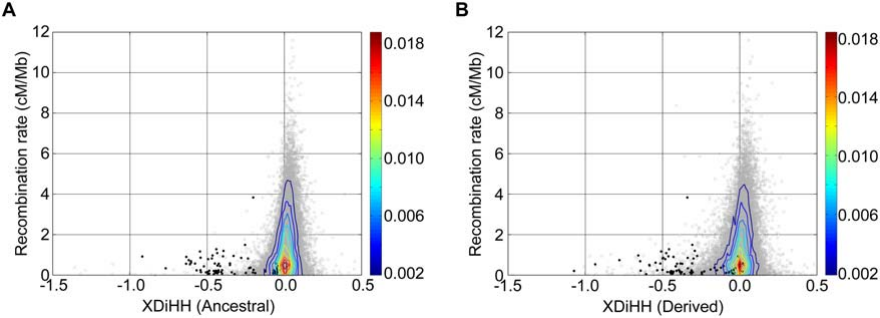
Relationship between XDiHH and fine-scale recombination rate. XDiHH (fastPHASEd-piCHM vs. CHM) of ancestral (A) and derived (B) alleles and fine-scale recombination rate from HapMap II were calculated in 100 kb windows (gray circles). The signals from MHC locus are in black circles. The color ranges for fraction are indicated in a side bar.

Figure 4 shows the overall distribution of XDiHH when plotted against the recombination rate [31]. As is shown in the figure, XDiHH of derived alleles is generally more negative than that of ancestral alleles (mean values −0.0315 vs. −0.0059). This is consistent with the fact that the derived alleles arose by new mutations and are typically associated with longer haplotypes, and are therefore more sensitive to fragmentation by phasing errors than are ancestral alleles [17],[32]. In addition, the majority of XDiHH values were in weakly positive areas, while clusters of extremely negative XDiHH values were observed in the low recombination rate range, where the MHC locus signal was observed. These are consistent with the expectation from the simulation results (see legend to Figure S2), and are consistent with the results of the local analysis of chromosome 6p by PHASE.

We also examined the genome-wide view of the XDiHH profile for CEU and YRI, which was calculated using the haplotypes inferred by fastPHASE in the absence of trio information. Regions showing exceptionally negative XDiHH values in CEU and YRI are listed in Tables S7 and S8. It is noteworthy that previously reported positively selected regions are significantly enriched in the regions of excessively negative XDiHH, possibly suggesting that regions under positive selection are more likely to have reduced XDiHH by phasing errors. We also noticed prominent negative values at 17q21.31 in CEU, but the corresponding region in YRI or CHM did not reveal such negative values. This site has been reported as a locus under selection specifically in Europeans, due to inversion polymorphism [33].

### Effects of Known Haplotypes on the Accuracy of Phasing Unrelated Individuals

The recent increase in large-scale association studies is yielding a high volume of high-density genotype data, and presently available haplotype maps are likely to be greatly improved by incorporating these newly collected data [5],[6],[9]. The question here is whether and how the availability of definitive haplotype data can improve the accuracy of inference of haplotypes of unrelated individuals.

To answer this question, we phased the data of the same sources by two procedures. One was phasing using only genotypes. In this procedure, combined datasets of JPT and/or CHB in HapMap II and all piCHMs (49 pairs) were used (simple phasing). Another was phasing genotypes with definitive haplotypes of CHMs serving as references (referenced phasing). In this procedure, we used the same JPT/CHB and a subset of piCHM (5 pairs) as genotypes to be phased, and the remaining CHM (88 haplotypes) served as a reference haplotype dataset. Phasing accuracy was evaluated by comparing the inferred haplotypes of the five piCHMs (that were common in all phasings) with the true haplotypes, employing Beagle program (ver. 3.0.1) [6], which is capable of phasing genotypes in the presence or absence of reference (or definitive) haplotypes.

**Figure 5:**
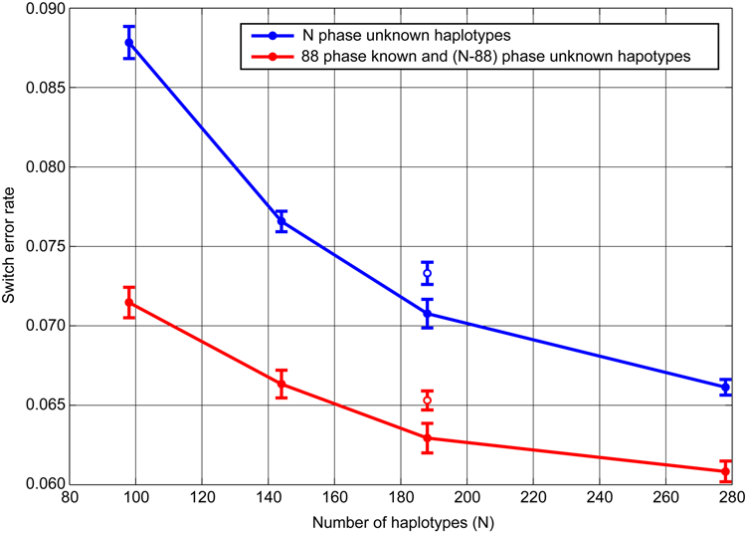
Effects of availability of true haplotypes on the switch error rate in the inference of phases. The filled blue circles show the switch error rates estimated for simple phasings using the genotypes of 49 piCHMs (all pairs) and various numbers of HapMap individuals (0, 23 JPTs, 45 JPTs, or 45 JPTs+45 CHBs). The filled red circles show the switch error rates for the referenced phasings using combined datasets, each consisting of a reference haplotype set (88 CHM haplotypes) and two genotype sets, which are 5 piCHMs (5 pairs made of a subset of CHM haplotypes not included in the reference set) and various numbers of HapMap individuals (0, 23 JPTs, 45 JPTs, or 45 JPTs+45 CHBs). Phasings were done using Beagle program (ver. 3.0.1) and the switch error rates were evaluated using the 5 pairs of piCHMs that were included in all datasets. For both phasings, the numbers of haplotypes participated in the inferences (N) were 98, 144, 188, and 278, respectively. The open circles at N = 188 show the switch error rates of a simple phasing using a genotype dataset of 49 piCHM and 45 CHBs (blue), and a referenced phasing using a combined dataset consisting of 88 CHM haplotypes, 5 piCHM genotypes, and 45 CHB genotypes (red). The results are in means and SDs of three trials using randomly paired different piCHM datasets.

As shown in Figure 5, the global switch error rate observed in the simple phasing of 49 piCHMs was approximately 0.0878 (evaluated using 5 piCHMs), which was somewhat higher than that expected from the reported value for Beagle [5]. This may be attributable to the difference in the nature of the data source (pseudo-individuals of real haplotypes vs. phased genotypes generated by population simulation). On the other hand, phasing with reference yielded significantly better inference haplotypes, that is, a switch error of 0.0715, when 88 reference haplotypes were used. When the number of unphased genotypes was increased (45 JPT and 45 CHB unphased genotypes were added), the accuracy of both phasings steadily improved, and the difference in the switch error levels between the two phasings gradually decreased. However, the phasing accuracy of Asian genotypes of unrelated individuals can be significantly improved if true haplotype data are included in the phasing procedures (Beagle), at least at the sample sizes employed by the HapMap Project II (0.0608 vs. 0.0661). Interestingly, the accuracy in the combined datasets of sub-structured populations (CHM+CHB) was significantly poorer than that of the non-structured populations (CHM+JPT), indicating that in order to improve the phasing accuracy by increasing the sample size, it is best to use samples of the same population.

## Discussion

We determined high-resolution, genome-wide definitive haplotypes of East Asians (Japanese) by genotyping haploid materials (CHMs) using Affymetrix 500 K array sets, and assessed the influence of non-Mendelian inference of haplotypes to downstream analyses across the entire genome. We employed a new statistical method, XDiHH, to detect the difference in the extent of haplotype homozygosity between two haplotype datasets. The assessment was made after restricting the analysis to only shared SNPs and matching sample sizes, in order to achieve a strict comparison between the datasets. We examined XDiHH between the haplotypes obtained by phasing pseudo-individuals and the original true haplotypes. We found that, in some regions, notably at the MHC locus, the XDiHH values were considerably lowered, regardless of the datasets of the true haplotypes or the program used for phasing (PHASE, fastPHASE, or Beagle), as long as the allele assignments were done in the absence of pedigree information.

The accuracy of phasing genotypes of unrelated individuals can be increased by increasing the number of individuals [6]. We have shown that the switch error rate of phasing using a reference panel of 88 known haplotypes was 0.0715, which is equivalent to the rate when 89 phase-unknown genotypes (i.e., genotypes for 178 halotypes) contributed to the phasing (Figure 5). Thus, the phased haplotypes were approximately two-times more efficient than the unphased data in the phasing by Beagle at the sample size examined. It is interesting to know how the fixed number of reference panel can contribute to the improvement of phasing accuracy, when the number of unphased individuals to be phased is further increased. However, it is likely that increasing the number of the definitive haplotypes in the reference panel can significantly improve the phasing accuracy even at sample size ranges far exceeding those examined here.

Recently, several next-generation technologies for DNA sequencing have become available or are being developed [34]–[37], and projects on whole genome resequencing of large numbers of individuals in diverse populations have been proposed or are now underway. These projects will bring us knowledge of the entire genetic make-up of humans, including rare forms of genetic variation. However, further laborious tasks, such as discrimination of heterozygous sites and haplotype assembly, remain to be solved. These tasks are especially difficult in the case of rare variations. Perhaps using either haploid cells such as CHMs or trio samples may alleviate the burden of downstream analyses arising from phasing uncertainty.

We are presently genotyping another set of SNPs (Affymetrix Genome-Wide Human SNP Array 6.0 and Illumina Human1M-Duo DNA Analysis BeadChip) using more than 100 CHM samples, which are expected to be useful as reference haplotypes as well as to develop or calibrate models for future accurate haplotype inference.

The data generated here are in D-HaploDB [11] and are freely accessible via the internet at http://finch.gen.kyushu-u.ac.jp.

## Materials and Methods

### Ethics Statement

Informed consent was obtained from all donors of the CHM tissues. Use of these samples in the present work was approved by the Ethical Committee of Kyushu University.

### CHM Samples, Genotyping, and Data Processing

CHM samples were collected in a nationwide effort supported by the Japan Association of Obstetricians and Gynecologists. Both the female donors and their male partners were self-reported Japanese. Genomic DNA was extracted using a QIAamp DNA Mini Kit (Qiagen).

DNA of 100 CHMs confirmed to have no heterozygous sites by microsatellite analyses were genotyped using Affymetrix GeneChip 500 K arrays (Santa Clara, California, United States; http://www.affymetrix.com). The physical coordinates of SNPs referenced to NCBI build36 of the human genome were obtained from the Affymetrix web site. The Dynamic Model at P = 0.33 was employed to call the alleles, and the concordance for the 50 SNPs that were common in the Nsp and Sty arrays was over 93.9%. The call rate of the worst CHM was 94.86% (Table S9), and the averaged sample call rate was 97.59%, while the averaged SNP call rate for all CHMs was 98.58% (Table S10).

A subset of the CHMs examined in the present study (74 of 100) had been genotyped by Perlegen SNP arrays (280 K SNPs) in our previous study [12], and 56,883 SNPs were re-typed in the present work using Affymetrix 500 K Array Sets. Based on the results of these shared typings, the concordance rate was calculated to be 99.96%. In principle, all genotype calls of CHM samples should be homozygous, since the materials are haploid. However, a small fraction (0.67%) of the calls was heterozygous. We believe that at least some, if not all, of these heterozygous calls were attributable to signals from the paralogous regions, and the rest were typing errors. In subsequent analyses, all heterozygous calls were considered to be “no calls,” since the changes were unlikely to seriously affect any of the conclusions described here. The raw microarray CEL and CHP files have been submitted to Gene Expression Omnibus database at NCBI (GEO; http://www.ncbi.nlm.nih.gov/geo) under accession number GSE12713.

### CHM Haplotype Datasets

To make it more convenient to compare datasets using various statistical methods, we adjusted the number of samples to 86, which was the maximum sample number available for JPTs after quality-checking (see below). CHM065 was excluded from the dataset for reasons described in the Results section, and 86 samples were chosen from the remainder based on their high call rate (>96.30%). We imputed the missing genotypes (3.08%, including those converted from heterozygote calls) using fastPHASE v.1.3.0 beta for Linux [8], so that the analyzed dataset did not contain any missing values. This version of the program was kindly provided by P. Sheet [8].

We also created inferred haplotype datasets by phasing pseudo-individuals (piCHMs) that were created by randomly pairing the CHM haplotypes. Haplotypes of chromosome 6p was inferred by PHASE (v2.1) using the default settings, after dividing the region into 25 non-overlapping segments, each with 500 SNPs. Neighboring segments of PHASEd-piCHM haplotypes were linked by referring to the phases of CHM at heterozygous sites that were nearest to the segment ends. This method of segment phasing is not as strict as that employed in PHASElink [9]. However, the present method does not overestimate the XDiHH values at the junction, because the phases of the two SNPs that bridge the segments are forced to be the same between PHASEd-piCHM and CHM, and the likelihood that the haplotypes branch at the junction is equal between the two datasets.

Genome-wide haplotypings of piCHMs were carried out using either fastPHASE v.1.3.0 beta for Linux [8] with ‘-KL6 -KU14 Ki2’ options or Beagle v.2.1.3 [5] with nsamples = 25 option.

### JPT Haplotype Datasets

For HapMap JPT samples, two individuals, NA18987 and NA18992, whose estimated cryptic relatedness coefficients have been shown to be more than 1/32 [2], were excluded from consideration in order to avoid inappropriate inflation of homozygosity statistics due to their unusually long-range haplotype homozygosity. PHASEd-JPT was a subset of the haplotype datasets in the HapMap database (http://www.hapmap.org/downloads/phasing/2007-08_rel22/), created by extracting the data for the SNPs that were genotyped using the Affymetrix 500 K Array.

### CEU Haplotype Datasets

Forty-three CEU individuals (parents) with high call rates (>99.08%) were selected for analyses. The cryptic relatedness coefficients for these samples were less than 1/32 [2]. The individual IDs used in this study are listed with their call rates in Supplemental Table S11. Haplotypes of chromosome 6p for all SNPs in HapMap Phase II using PHASE (v2.1) in the absence of offspring information were inferred using the default settings, after dividing the region into 138 non-overlapping segments, each carrying 500 SNPs. Neighboring segments of PHASEd-CEU haplotypes were linked by referring to the HapMap data (i.e., phases of PHASEd+CEU) as described above.

### YRI Haplotype Datasets

Forty-three YRI individuals (parents) with high call rates (>99.06%) were selected for analyses. The cryptic relatedness coefficients for these samples were less than 1/32 [2]. The individual IDs used in this study are listed with their call rates in Table S11.

### Principal Component Analysis

We applied principal component analysis (PCA) to detect hidden population stratification by using the SMARTPCA program of the EIGENSOFT version 2.0 package [19],[20]. For this analysis, the CHM haplotypes were randomly paired to create pseudo-individuals. Figures 1 and S1 were generated using different pairs. PCA was applied using autosomal SNPs, for which more than 99% of the samples were successfully genotyped [1].

### Obtaining Ancestral States from Alignment of Human and Chimpanzee Sequences

To obtain ancestral states for SNPs, we obtained alignment sequence files between human and chimpanzee sequences from the UCSC database (http://hgdownload.cse.ucsc.edu/goldenPath/hg18/vsPanTro2/axtNet/). We then assumed the chimpanzee allele at the appropriate position to be ancestral. For 97.9% of SNPs (479,864 of 489,992), the ancestral states were successfully determined. No ancestral state was inferred for the remainder.

### iHS Calculation

The iHH was computed as described in previous work, using the SNPs with minor allele frequencies greater than 5% [17]. For both ancestral and derived chromosomes, we calculated EHH values between the core SNP and every other SNP and integrated with respect to genetic distance over the longest region for which at least two haplotypes were homozygous. The genetic distance data were downloaded from the HapMap database (corrected on June 30, 2008) [1]. These integrals were denoted as iHH_Ancestral_ or iHH_Derived_. If the region spanned by EHH reached gaps was longer than 500 kb or reached the chromosome ends, no further iHH scoring was reported for the core SNP. The unstandardized integrated haplotype score, which is ln(iHH_Ancestral_/iHH_Derived_), was then calculated for every SNP. The scores were standardized for the whole genome by normalizing them according to the frequency of the derived allele, in order to obtain iHS. These normalized scores have zero mean and unit variance.

### Switch Error Rate

The rate is defined as sw/(n – 1), where n denotes the number of heterozygous sites and sw is the number of switches between neighboring heterozygous sites needed to recover the original haploid sequence [29],[30]. In computing these scores for each individual, we ignored sites where one or both alleles were missing.

## Supporting Information

Figure S1 Principal component analysis of CHM and HapMap samples. Plots of the first two eigenvectors for three Asian datasets (A and C) and all five datasets of three ethnicities (B and D) are shown. The population panels were from the HapMap Project. The CHM haplotypes were randomly paired to create pseudo-individuals. Pairs different from those in (A and B) were subjected to analysis in (C and D). Autosomal SNPs with less than 99% complete genotyping were filtered out, leaving 289,565 (A), 306,146 (B), 289,548 (C) and 306,111 (D) SNPs. One JPT sample (NA18976) appears to have mixed ancestry, which is consistent with a previous report by the HapMap Project [1].
(1.09 MB TIF)

Figure S2 Simulation of the effect of switch error on XDiHH under various LD conditions. We simulated simplified cases of two SNP loci, both heterozygous for 50 individuals. There are only two possible genotypes, (0,0)/(1,1) and (0,1)/(1,0). Under various LD conditions (r^2^ between 0 and 1), the ratio of the two genotypes are uniquely determined. These consist of genotype datasets with true phases. We then introduced all possible switches (from 0 to 50) to make genotype datasets of switched phases, and XDiHH values were calculated against the true values for each trial, according to the equation given in the text. The XDiHH were then plotted against the switch error rate for each r^2^ value, as shown in the figure. In this simulation, both the switch error rate and the XDiHH values are discrete, and each dot in the figure denotes only the possibility of occurrence in this space and does not represent the frequency. The figure indicates that in the genomic regions of high r^2^ (or low recombination rate), the XDiHH value tends to decrease.
(1.11 MB TIF)

Figure S3 Fine-scale XDiHH map of chromosome 6p in CEU. From top to bottom panel, the XDiHH values of ancestral and derived alleles of PHASEd-CEU vs. PHASEd+CEU, switch error (sw) rate, and recombination rate. See Figure 3 legend for further details.
(0.87 MB TIF)

Table S1 ANOVA statistics for population differences along first two eigenvectors.
(0.02 MB XLS)

Table S2 Differential enrichment of iHS outliers.
(0.02 MB XLS)

Table S3 Candidate regions for recent selection from CHM datasets by iHS test.
(0.10 MB XLS)

Table S4 Candidate regions for recent differential selection by XP-EHH test between CHM and CEU.
(0.05 MB XLS)

Table S5 Candidate regions for recent differential selection by XP-EHH test between CHM and YRI.
(0.06 MB XLS)

Table S6 Summary of regions ranked by XDiHH (fastPHASEd-piCHM vs. CHM).
(0.06 MB XLS)

Table S7 Summary of regions ranked by XDiHH (fastPHASEd-CEU vs. PHASEd+CEU).
(0.06 MB XLS)

Table S8 Summary of regions ranked by XDiHH (fastPHASEd-YRI vs. PHASEd+YRI).
(0.07 MB XLS)

Table S9 Call rate for CHM.
(0.03 MB XLS)

Table S10 Distribution of missing genotypes among 100 CHMs.
(0.02 MB XLS)

Table S11 HapMap samples used in this study.
(0.02 MB XLS)

## References

[1] Frazer KA, Ballinger DG, Cox DR, Hinds DA, Stuve LL (2007). A second generation human haplotype map of over 3.1 million SNPs.. Nature.

[2] The International HapMap Consortium (2005). A haplotype map of the human genome.. Nature.

[3] Levy S, Sutton G, Ng PC, Feuk L, Halpern AL (2007). The diploid genome sequence of an individual human.. PLoS Biol.

[4] Stephens M, Scheet P (2005). Accounting for decay of linkage disequilibrium in haplotype inference and missing-data imputation.. Am J Hum Genet.

[5] Browning SR, Browning BL (2007). Rapid and accurate haplotype phasing and missing-data inference for whole-genome association studies by use of localized haplotype clustering.. Am J Hum Genet.

[6] Browning BL, Browning SR (2009). A unified approach to genotype imputation and haplotype-phase inference for large data sets of trios and unrelated individuals.. Am J Hum Genet.

[7] Eronen L, Geerts F, Toivonen H (2006). HaploRec: efficient and accurate large-scale reconstruction of haplotypes.. BMC Bioinformatics.

[8] Scheet P, Stephens M (2006). A fast and flexible statistical model for large-scale population genotype data: applications to inferring missing genotypes and haplotypic phase.. Am J Hum Genet.

[9] Marchini J, Cutler D, Patterson N, Stephens M, Eskin E (2006). A comparison of phasing algorithms for trios and unrelated individuals.. Am J Hum Genet.

[10] Andres AM, Clark AG, Shimmin L, Boerwinkle E, Sing CF (2007). Understanding the accuracy of statistical haplotype inference with sequence data of known phase.. Genet Epidemiol.

[11] Higasa K, Miyatake K, Kukita Y, Tahira T, Hayashi K (2007). D-HaploDB: a database of definitive haplotypes determined by genotyping complete hydatidiform mole samples.. Nucleic Acids Res.

[12] Kukita Y, Miyatake K, Stokowski R, Hinds D, Higasa K (2005). Genome-wide definitive haplotypes determined using a collection of complete hydatidiform moles.. Genome Res.

[13] Fan JB, Surti U, Taillon-Miller P, Hsie L, Kennedy GC (2002). Paternal origins of complete hydatidiform moles proven by whole genome single-nucleotide polymorphism haplotyping.. Genomics.

[14] Taillon-Miller P, Bauer-Sardina I, Zakeri H, Hillier L, Mutch DG (1997). The homozygous complete hydatidiform mole: a unique resource for genome studies.. Genomics.

[15] Sabeti PC, Reich DE, Higgins JM, Levine HZ, Richter DJ (2002). Detecting recent positive selection in the human genome from haplotype structure.. Nature.

[16] Sabeti PC, Varilly P, Fry B, Lohmueller J, Hostetter E (2007). Genome-wide detection and characterization of positive selection in human populations.. Nature.

[17] Voight BF, Kudaravalli S, Wen X, Pritchard JK (2006). A map of recent positive selection in the human genome.. PLoS Biol.

[18] Tang K, Thornton KR, Stoneking M (2007). A new approach for using genome scans to detect recent positive selection in the human genome.. PLoS Biol.

[19] Patterson N, Price AL, Reich D (2006). Population structure and eigenanalysis.. PLoS Genet.

[20] Price AL, Patterson NJ, Plenge RM, Weinblatt ME, Shadick NA (2006). Principal components analysis corrects for stratification in genome-wide association studies.. Nat Genet.

[21] Johnson GC, Esposito L, Barratt BJ, Smith AN, Heward J (2001). Haplotype tagging for the identification of common disease genes.. Nat Genet.

[22] Carlson CS, Eberle MA, Rieder MJ, Yi Q, Kruglyak L (2004). Selecting a maximally informative set of single-nucleotide polymorphisms for association analyses using linkage disequilibrium.. Am J Hum Genet.

[23] Servin B, Stephens M (2007). Imputation-based analysis of association studies: candidate regions and quantitative traits.. PLoS Genet.

[24] Marchini J, Howie B, Myers S, McVean G, Donnelly P (2007). A new multipoint method for genome-wide association studies by imputation of genotypes.. Nat Genet.

[25] Conrad DF, Jakobsson M, Coop G, Wen X, Wall JD (2006). A worldwide survey of haplotype variation and linkage disequilibrium in the human genome.. Nat Genet.

[26] de Bakker PI, McVean G, Sabeti PC, Miretti MM, Green T (2006). A high-resolution HLA and SNP haplotype map for disease association studies in the extended human MHC.. Nat Genet.

[27] Tishkoff SA, Reed FA, Ranciaro A, Voight BF, Babbitt CC (2007). Convergent adaptation of human lactase persistence in Africa and Europe.. Nat Genet.

[28] Bersaglieri T, Sabeti PC, Patterson N, Vanderploeg T, Schaffner SF (2004). Genetic signatures of strong recent positive selection at the lactase gene.. Am J Hum Genet.

[29] Stephens M, Donnelly P (2003). A comparison of bayesian methods for haplotype reconstruction from population genotype data.. Am J Hum Genet.

[30] Lin S, Cutler DJ, Zwick ME, Chakravarti A (2002). Haplotype inference in random population samples.. Am J Hum Genet.

[31] McVean GA, Myers SR, Hunt S, Deloukas P, Bentley DR (2004). The fine-scale structure of recombination rate variation in the human genome.. Science.

[32] Watterson GA, Guess HA (1977). Is the most frequent allele the oldest?. Theor Popul Biol.

[33] Stefansson H, Helgason A, Thorleifsson G, Steinthorsdottir V, Masson G (2005). A common inversion under selection in Europeans.. Nat Genet.

[34] Eid J, Fehr A, Gray J, Luong K, Lyle J (2009). Real-time DNA sequencing from single polymerase molecules.. Science.

[35] Margulies M, Egholm M, Altman WE, Attiya S, Bader JS (2005). Genome sequencing in microfabricated high-density picolitre reactors.. Nature.

[36] Metzker ML (2005). Emerging technologies in DNA sequencing.. Genome Res.

[37] Shendure J, Porreca GJ, Reppas NB, Lin X, McCutcheon JP (2005). Accurate multiplex polony sequencing of an evolved bacterial genome.. Science.

