# Supplementary figures and images for: Evaluation of Haplotype Inference Using Definitive Haplotype Data Obtained from Complete Hydatidiform Moles, and Its Significance for the Analyses of Positively Selected Regions
Source: PLoS Genet. 2009 May 8;5(5):e1000468. doi: 10.1371/journal.pgen.1000468 (PMC2670534; doi:10.1371/journal.pgen.1000468)

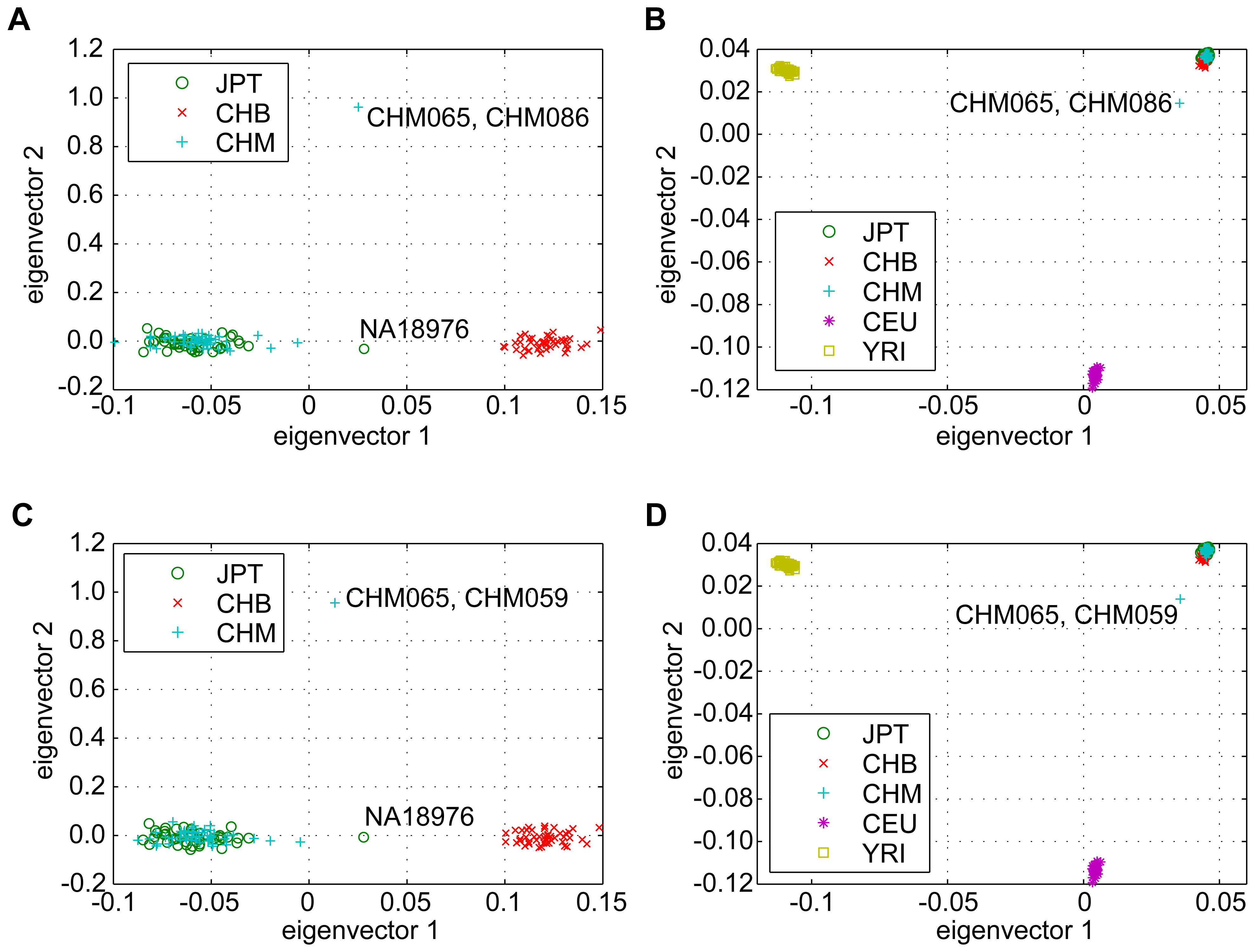

Supplement: Figure S1 — Principal component analysis of CHM and HapMap samples. Plots of the first two eigenvectors for three Asian datasets (A and C) and all five datasets of three ethnicities (B and D) are shown. The population panels were from the HapMap Project. The CHM haplotypes were randomly paired to create pseudo-individuals. Pairs different from those in (A and B) were subjected to analysis in (C and D). Autosomal SNPs with less than 99% complete genotyping were filtered out, leaving 289,565 (A), 306,146 (B), 289,548 (C) and 306,111 (D) SNPs. One JPT sample (NA18976) appears to have mixed ancestry, which is consistent with a previous report by the HapMap Project [1]. (1.09 MB TIF) [file pgen.1000468.s001.tif]

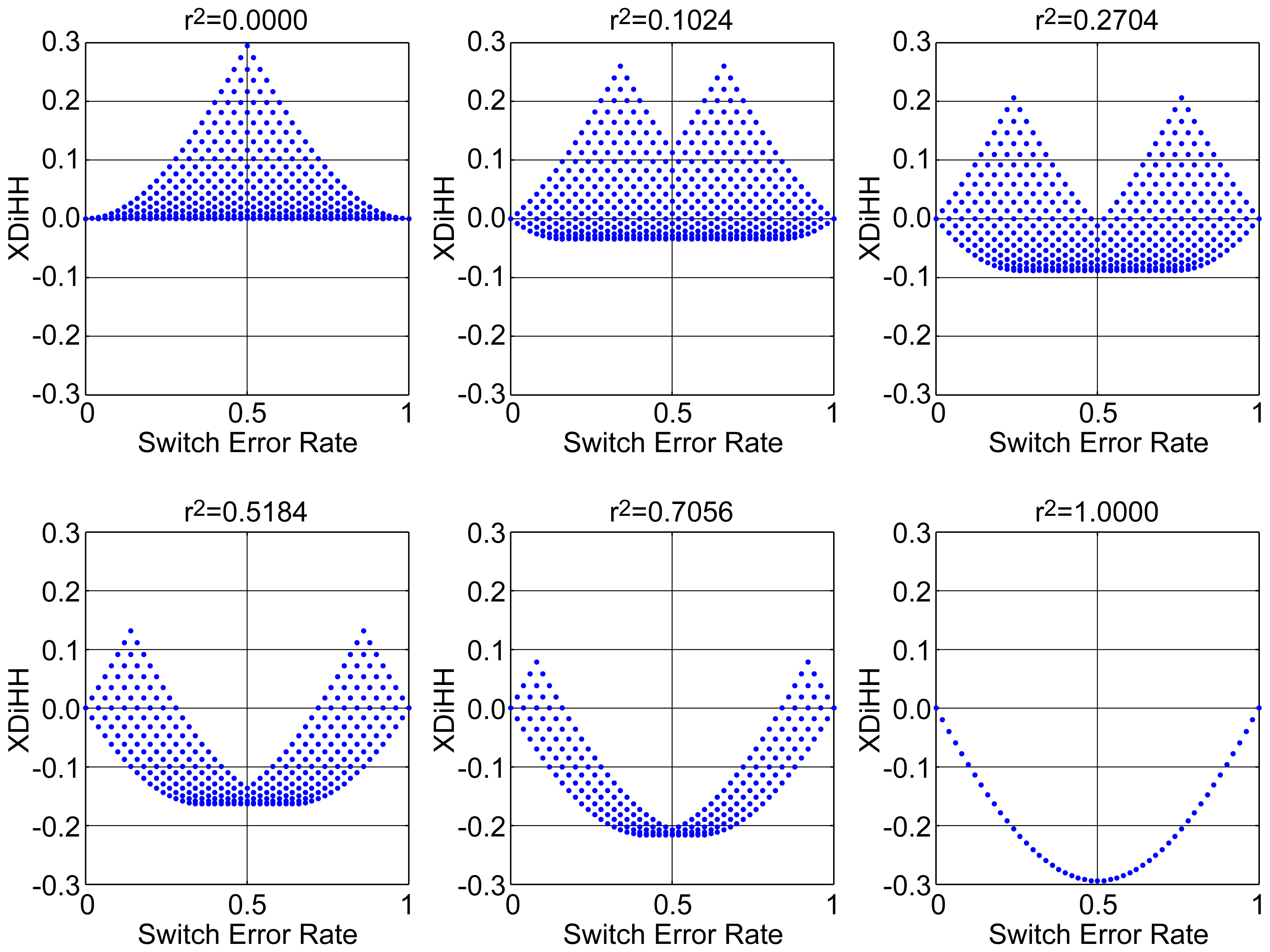

Supplement: Figure S2 — Simulation of the effect of switch error on XDiHH under various LD conditions. We simulated simplified cases of two SNP loci, both heterozygous for 50 individuals. There are only two possible genotypes, (0,0)/(1,1) and (0,1)/(1,0). Under various LD conditions (r2 between 0 and 1), the ratio of the two genotypes are uniquely determined. These consist of genotype datasets with true phases. We then introduced all possible switches (from 0 to 50) to make genotype datasets of switched phases, and XDiHH values were calculated against the true values for each trial, according to the equation given in the text. The XDiHH were then plotted against the switch error rate for each r2 value, as shown in the figure. In this simulation, both the switch error rate and the XDiHH values are discrete, and each dot in the figure denotes only the possibility of occurrence in this space and does not represent the frequency. The figure indicates that in the genomic regions of high r2 (or low recombination rate), the XDiHH value tends to decrease. (1.11 MB TIF) [file pgen.1000468.s002.tif]

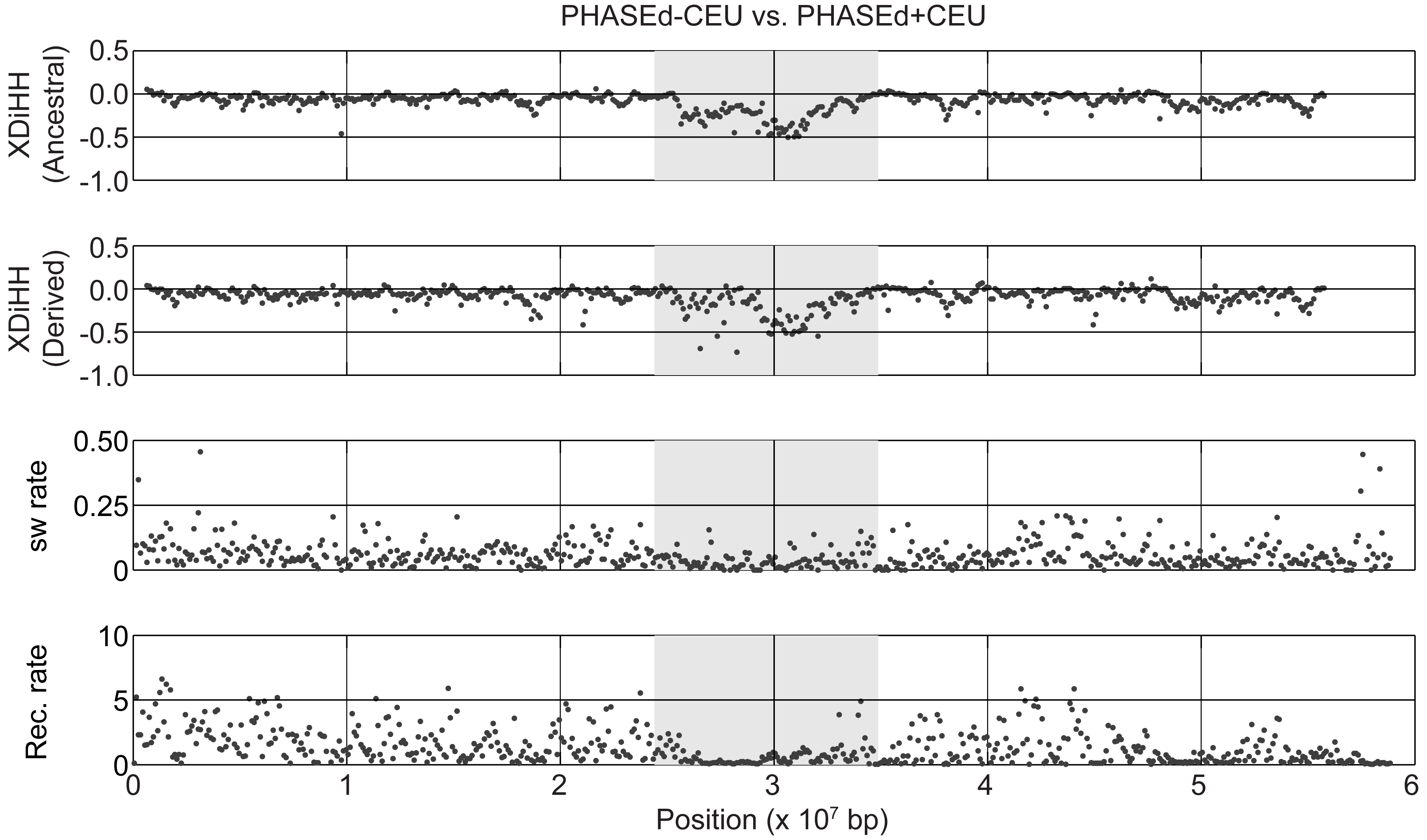

Supplement: Figure S3 — Fine-scale XDiHH map of chromosome 6p in CEU. From top to bottom panel, the XDiHH values of ancestral and derived alleles of PHASEd-CEU vs. PHASEd+CEU, switch error (sw) rate, and recombination rate. See Figure 3 legend for further details. (0.87 MB TIF) [file pgen.1000468.s003.tif]
